# Magnitude of kangaroo mother care practice and its associated factors in Tigray region, northern Ethiopia, 2019: cross-sectional study design

**DOI:** 10.11604/pamj.2023.44.5.29894

**Published:** 2023-01-04

**Authors:** Ebud Ayele, Hagos Tasew, Teklewoini Mariye, Guesh Gebreayezgi, Degena Bahrey, Kiros Gereziher, Shewit Engdashet, Tsehaynesh Gidey, Aregawi Gebreyesus

**Affiliations:** 1 Department of Public Health Nutrition, College of Medicine and Health Sciences, Axum University, Axum, Ethiopia,; 2 Department of Nursing, College of Medicine and Health Science, Axum University, Axum, Ethiopia,; 3 Department of Public Health Epidemiology, College of Medicine and Health Sciences, Axum University, Axum, Ethiopia,; 4 Department of Public Health Economics, College of Medicine and Health Science, Mekelle University, Mekelle, Ethiopia,; 5 Department of Public Health Epidemiology, College of Medicine and Health Science, Mekelle University, Mekelle, Ethiopia

**Keywords:** Kangaroo mother care, practice, Tigray, Ethiopia

## Abstract

**Introduction:**

kangaroo mother care is an evidence based approach care of preterm and low birth weight infants carried skin-to-skin with the parents that can decrease morbidity and mortality of infant. Country level adoption and practice of kangaroo mother care has been limited and global coverage remains low and few studies have examined the reasons for low practice. The aim of this study was to assess the magnitude of kangaroo mother care practice and its associated factors in Tigray, northern Ethiopia, 2019.

**Methods:**

an institutional-based cross-sectional study design was conducted in public general hospitals of Tigray, northern Ethiopia, 2019. Two-stage sampling technique was used and an interviewer-administered questionnaire were used to collect the necessary information. The data were cleaned and entered using epi-Data version 3.1 then exported to stoical package for social science (SPSS) version 22.0 for analysis. Bivariate logistic regression and multivariable analysis were carried out at adjusted odds ratios (AOR) with 95% CI and significance level p-value (<0.05).

**Results:**

out of the total 844 selected mothers with their infants, 840 were participated in the study yielding to a response rate of 99.5%, of these respondent’s kangaroo mother care practice was found to be 70.2%. Being mothers housewife [(AOR=4.12, 95% CI: (1.5, 0.11)], maternal age [(AOR=9.3, 95% CI: (2.5, 33.9 )], currently mode delivery [(AOR=5.39, 95% CI: (2.3, 12.25)], number of children [(AOR=8.38, 95%: (4.6, 15.3)], mother having ≥5 children [(AOR=18.2, 95%CI: (9.4, 35.4)], antenatal care [(AOR=3.299 95%CI: (1.54, 7.07)] were factors at p-value (<0.05) significantly associated with kangaroo mother care practice.

**Conclusion:**

in this study, maternal age, parity, antenatal care, occupation and mode of delivery were factors that influence kangaroo mother care practice in the study area, so healthcare providers, policymakers and other stakeholders should give special focuses on those influencing factors.

## Introduction

Kangaroo mother care (KMC) is an infant carried skin-to-skin care with the mother in preterm and low birth weight infants [1,2], and is an intervention aimed at improving outcomes among preterm and low birth weight newborns [3]. Adequately implement and effectively scale-up of this intervention have a positive impact on the health of mothers and infants [1]. Hospitals and birthing centers have found it difficult to develop policies that will allow skin-to-skin care and rooming-in to continue safely [4]. After birth, separation of mothers and infants seems to be common practice in many hospitals [5].

According to the World Health Organization globally, more than 2.7 million newborns die each year, accounting for 44% of children dying before the age of five years [6]. Studies have depicted that kangaroo mother care can reduce in infant mortality rate at 3, 6 or 12 months by 41% [3], and have a positive impact on the health of mothers in certain cases, including early breastfeeding, early discharge from the healthcare facility [7]. According to the World health organization, kangaroo mother care promotes exclusive breastfeeding, bonding and attachment [8]. Moreover, kangaroo mother care was found to increase weight, length and head circumference, So these better growth results could reduce the morbidity and mortality as well [9]. The clinical efficacy and health benefits of kangaroo mother care have been demonstrated in multiple settings [10]. Country-level adoption and practice of kangaroo mother care has been limited and global coverage remains low and few studies have examined the reasons for low practice [1,1]. Such studies are important in developing countries, like Ethiopia to assess the present image of kangaroo mother care practice, there is limited study conducted in Tigray, regarding kangaroo mother care practice; and the available literatures in Ethiopian were limited in addressing factories that influence kangaroo mother care practice among mothers delivered, therefore, this study aims to assess magnitude and factors associated with kangaroo mother care practice among mothers delivered in a public general and referral hospitals of Tigray, northern Ethiopia.

## Methods

**Study design:** hospital-based cross-sectional study design was employed in public general hospitals of Tigray region, Ethiopia

**Study setting and population:** the study was carried out in public general hospitals of Tigray region, Tigray regional State is located around 780 kilometers north of the Ethiopian capital Addis Ababa, and with an elevation of 2,254 meters above sea level. Five public general hospitals Mekelle Hospital, St. Marry hospital, Lemlem Karl Hospital, Kahsay Abera Hospital and Adigrat Hospital were the selected study area. Data collection for this study was undertaken from August 01 to December 30/2019. The source populations were all mothers with their preterm and low birth weight neonate admitted to public general hospitals. Selected mothers with their preterm and low birth weight neonate admitted to general hospitals of Tigray, Ethiopia were the study population. All selected mothers with their preterm and low birth weight neonate delivered in public general hospitals during the study period were included; whereas, the unconscious and mother delivered by cesarean section were excluded.

**Variables:** socio-demographic characteristics (mothers´ age, maternal educational status, occupation, residence, marital status, income, religion and husband education) and maternal healthcare factors (parity, mode of delivery, complication during delivery, antenatal care (ANC) follow-up, any illness during pregnancy and having previous information on kangaroo mother care) were the dependent variable. Whereas kangaroo mother care implementation was the independent variable.

### Data collection resource and measurement

**Data collection tool:** structured questionnaire initially prepared in English and then translated into local language, Tigrigna were used. Tigrigna version was again translated back to English to check for any inconsistencies or distortion in the meaning of words. Data was collected using observation and interviewer administered structured questionnaire adapted from literatures [1,2,13].

**Data collection:** socio-demographic characteristics and maternal healthcare were the two main sections of dependent variable. Data collection was performed by Seven B.Sc. nurses, who were three day trained regarding the tool and the procedure. Continuous follow-up and supervision were made by two supervisors and principal investigator throughout the data collection period. The questionnaire was pre tested prior to the actual data collection on ten percent that were not included in the main data collection at Suhul public general hospital to check consistency of the questionnaire. The collected data were reviewed and checked for completeness by supervisors and principal investigator each week.

**Sampling size:** the sample size was calculated using single population proportion formula:


$$n=\frac{{\left(Z_{a/2}\right)}^{2}pq}{d^{2}}$$

By assuming; precision (d)= 5%, Confidence level=95% (Z_α/2_=1.96), the proportion of kangaroo mother care= 50%. Hence, the sample size by considering 10% non-response rate was 422. Because of the two stage sampling employed the design effect two (2*422) was used. Finally, 844 mothers were taken as a final sample size. In Tigray region, there were 14 public general hospitals, five public general hospitals were selected randomly and the sample size was proportionally allocated to each hospital. The systematic random sampling technique was used to select every (determined interval K=2) study subjects from each five hospitals.

**Data analysis:** after the data was entered in to the Epi-Data 3.1 then exported to (SPSS) Version 23 for analysis. Binary logistic regression analysis was executed to see the association between independent and outcome variables. All explanatory variables associated with the outcome variable with p < 0.25 were entered into multivariable logistic regression analysis and significant association was identified based on p < 0.05 and AOR with 95% CI. The operational definition of the study was as follows; kangaroo mother care practice: is care of preterm and low birth weight infants, whereby the infant is placed and held in direct skin-to-skin contact on the mother's chest in an upright position under her clothes [10,14]. Continuous kangaroo mother care: Is defined as the practice of skin-to-skin care continuously throughout the day without breaking the contact between mother and baby [15]. Intermittent kangaroo mother care: is the practice of skin-to-skin care alternated with the use of either a radiant warmer or incubator care for the baby [15].

**Ethics consideration:** ethical clearance was obtained from institutional review board (IRB) of Aksum University, college of health science. Official permission was secured from Tigray Region Health Bureau (TRHB) and respected hospitals. The respondents were informed about the objective and purpose of the study and written consent was obtained from each respondent during data collection. Confidentiality of the information was assured. Consent was obtained from their parents, for those who were less than 18 years old participant mothers. Respondents were allowed to refuse, discontinue or participate at any time they want.

## Results

**Socio-demographic characteristics:** a total of 840 mothers with an infant were included in the study with a response rate of 99.5%. The mean age of the mothers was 36(SD ± 1.3) years. Seven hundred seven (88.9%) of them were married, 268 (31.9%) of them were mothers doing agriculture, 772 (91.9%), of the mothers, were of Tigray ethnicity and 794 (71.2%) were orthodox in religion. Out of 747 married mothers, three hundred forty-two (40.7%) of their husbands had college and above education, 322 (38.3%) mothers were elementary school, and 280(33.3%) mothers were monthly income less than 1000 Ethiopian Birr (Table 1).

**Table 1:** socio-demographic characteristics of mothers with infants in public general hospitals of Tigray, Ethiopia, 2019 (n=840)

Variable	Frequency	Percent (100%)
**Marital status**		
Divorced	93	11.1
Married	747	88.9
**Occupation**		
Non-government employ	116	13.8
Private employ	231	27.5
Agriculture	268	31.9
Housewife	58	6.9
Government employ	167	19.9
**Religion**		
Orthodox	794	94.5
Muslim	46	5.5
**Mothers age**		
15-19	48	5.8
20-24	36	4.3
25-29	42	5.0
30-34	280	33.3
35-39	223	26.5
40-44	154	18.3
45-49	57	6.8
**House bands education**		
No education	58	6.9
Elementary	101	12.0
High school	246	29.3
College and above	342	40.7
Ethnicity		
Tigray	772	91.9
Other	68	8.1
**Mothers education**		
No education	142	16.9
Elementary	322	38.3
High school	268	31.9
college and above	108	12.9
**Income**		
1000 or less	280	33.3
1001-2000	225	26.8
2001-3000	246	29.3
3001 and above	89	10.6

**Figure 1:**
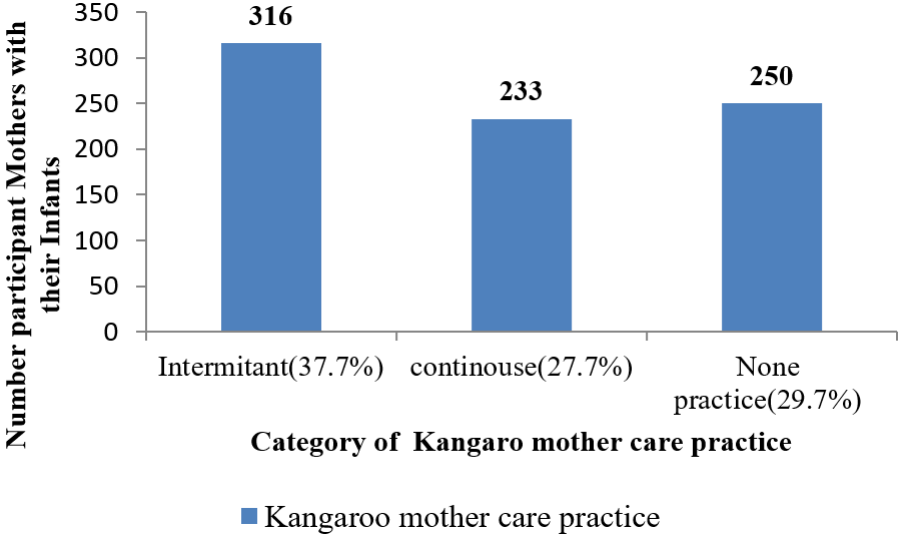
kangaroo mother care practice characteristics of mothers with infants in public general hospitals of Tigray, Ethiopia, 2019

**Maternal healthcare characteristics:** out of 840 mothers, about 590 (70.2%) were practiced kangaroo mother care, 652 (77.6%) had one-time antenatal care visit when they were pregnant, 676 (80.7%) out of mothers no history of illness during pregnancy, 595 (70.8%) were heard information about kangaroo mother care, 449 (59.4%) mothers were spontaneous vaginal delivered, 226 (26.9%) mothers were having 2-4 children, and about 710 (84.6%) mothers were haven't complication during delivery (Table 2). From the total participated women delivered 316 (37.6%) intermittent, 233 (27.7%) continuous kangaroo mother care was practiced (Figure 1).

**Table 2:** maternal health care characteristics of mothers with infants in public general hospitals of Tigray, Ethiopia, 2019 (n=840)

Variable	Frequency	Percentage (%)
**Illness during pregnancy**		
Yes	164	19.5
No	676	80.5
**Antenatal follow**		
1	652	77.6
2-3	188	22.4
**Type current delivery**		
Forceps	59	7.0
Vacuum	282	33.6
Spontaneous vaginal delivery	499	59.4
**KMC implementation**		
Yes	590	70.2
No	250	29.8
**Having information Kangaroo Mother Care (KMC)**		
Yes	595	70.8
No	245	29.2
**Parity**		
Prim Para	159	19
2-4 chidren	226	26.9
Multi Para ≥5	435	51.9
**Complication during delivery**		
Yes	130	15.4
No	710	84.6

**Bivariate analysis:** the bivariate analysis showed that marital status, maternal educational status, occupation, illness during pregnancy, information, antenatal care, follow-up, maternal age, type of current delivery, parity, income, and complications during delivery were crudely associated at 25% level of significance.

**Multivariate analysis:** mothers housewife were less likely [(AOR=0.20, 95% CI: (0.08, 0.54)] to practice kangaroo mother care than those who works in government employee, pregnant women 2-3 times visiting antenatal care were more likely [(AOR=3.299, 95% CI: (1.54, 7.07)] to practice kangaroo mother care than one time, maternal age at 40-44 were more likely [(AOR=4.12, 95% CI: (1.5, 0.11 )], 45-49 [(AOR=9.3, 95% CI: (2.5, 33.9 )] to practice kangaroo mother care than those at 15-19 years age group, current spontaneous vaginal delivery were more likely [(AOR=5.39, 95% CI: (2.3, 12.25)] to practice kangaroo mother care than those who were delivered by forceps and mothers having 2-4 children were more likely [(AOR=8.38, 95%: (4.6, 15.3)], mother having ≥5 children [(AOR=18.2, 95%: (9.4, 35.4)] to practice kangaroo mother care than Prim-Para (Table 3).

**Table 3:** factors associated with Kangaroo Mother Care (KMC) practice among mothers delivers in public general hospitals of northern Ethiopia, 2019 (n=840)

Variables	KMC practice		COR 95% CI	AOR 95% CI
	Yes	No		
**Marital’ status**				
Divorced	57	36	Ref	Ref
Marred	533	214	1.57(1.007, 25)	0.879(0.46, 1.68)
**Occupation**				
Non-governmental employ	93	23	Ref	Ref
Private employ	181	50	0.89(0.51, 1.6)	0.76(0.36, 1.6)
Agriculture	141	127	0.27(0.16, 0.46)	0.56(0.27, 1.13)
Housewife	23	34	0.16(.081, 0.3)	0.202(0.08, 0.54)**
Governmental employ	152	15	2.5(1.2, 5.0)	1.062(0.43, 2.6)
**Illness during pregnancy**				
Yes	104	60	Ref	Ref
No	486	190	1.476 (1.03, 2.1)	1.38(0.8, 2.35)
**Information**				
Yes	404	191	Ref	Ref
No	186	59	1.49(1.06, 2.09)	0.62(0.29, 1.34)
**Antenatal follow**				
1	416	236	Ref	Ref
2-3	174	14	7 (3.9, 12.4)	3.299(1.54, 7.07)*
**Maternal age**				
15-19	28	20	Ref	Ref
20-24	22	14	1.12(0.46, 2.7)	0.82(0.2, 2.8)
25-29	32	10	2.28(0.9, 5.7)	1.6(0.46, 5.5)
30-34	200	80	1.8(.9, 3.3)	2.157(0.9, 5.17)
35-39	142	81	1.2(.7, 2.3)	2.15(0.8, 5.4)
40-44	124	30	2.9(1.47, 5.9)	4.121(1.5, 11)*
45-49	42	15	2.(0.9, 4.5)	9.3(2.5, 33.9)**
**Type current delivery**				
Forceps	16	43	Ref	Ref
Vacuum	134	148	2.433 (1.3, 4.5)	0.97(0.4, 2.14)
Spontaneous vaginal delivery	440	59	20(10.6, 37.82)	5.39(2.3, 12.25)**
**Parity**				
Prime-Para	31	128	Ref	Ref
2-4 children	165	81	8.4(5.2, 13.5)	8.38(4.6, 15.3)**
Multiple ≥5	394	41	39.7(23.9, 65.9)	18.2(9.4, 35.4)**
**Mother education**				
No education	81	61	Ref	Ref
Elementary	212	110	1.451 (0.9, 2.17)	2.2(1.2, 4)
high school	207	61	2.5 (1.6, 3.9)	3.9(1.8, 8.5)
college and above	90	18	3.8(2.055, 6.9)	11.4(3.8, 34.19)
**Income**				
1000 or less	216	64	Ref	Ref
1001-2000	149	76	0.58(0.39, 0.86)	0.5(0.28, 0.9)
2001-3000	78	168	0.64(0.4, 0.94)	0.7(0.39, 1.237)
3001 and above	32	57	0.53(0.31, 0.883)	0.4(0.18, 0.92)
**Complication during delivery**				
Yes	9	121	Ref	Ref
No	241	469	0.15(0.07, 0.290)	0.35(0.13, 0.92)

P=<0.001**, p<0.05*

## Discussion

In this study finding, the magnitude of kangaroo mother care practice was 70.2% with a 95% CI of (67%, 73%); which is lower than the study conducted in Uganda [16], and Dessie referral hospital [17]; in which (75%, and 80.8% ) of participates kangaroo mother care practice respectively. however, this finding is higher than the studies conducted in Nigeria [12] and in a public institution in Ethiopia [18], which showed that ( 53.5%, 34.4%, and 28.1%) of kangaroo mother care practices respectively. The possible reason for this difference might be due to participating in social demographic characteristics and the promotion of different facilities for kangaroo mother care. Furthermore, this discrepancy might be also due to differences in the availability and accessibility of materials, in a safe health service environment.

In this study, housewives, mothers, maternal age 40-44 and 45-49 years, spontaneous vaginal current delivery, mothers having 2-4 children above, and pregnant women with ANC 2-3 visits were found to be significantly associated with kangaroo mother care practice. In this regard, housewife mothers were 80% less likely [(AOR=0.20, 95% CI: (0.08, 0.54)] to implement kangaroo mother care than those who work as government employees. This finding is similar to the study done in Aksum [19]. The reason might be mothers working as government employees are exposed to information, media, and repeated contact with different people. So they might have the confidence to practice kangaroo mother care.

This study revealed that maternal age at 40-44 was 4.12 more likely [(AOR=4.12, 95% CI: (1.5, .11)], 45-49 age 9.3 times [(AOR=9.3, 95% CI: (2.5, 0.33.9)] to practice kangaroo mother care than those at age 15-19 group. This was similar to the finding in Tanzanian and Singapore [20]. This is because younger mothers have unsatisfactory antenatal care, are less educated, and have inadequate prenatal care and fewer social supports than older mothers due to that younger mothers don´t practice kangaroo mother care more than older mothers. Current spontaneous vaginal delivery was 5.39 times more likely [(AOR=5.39, 95% CI: (2.3, 12.25)] to practice in kangaroo mother care than those who were delivered by forceps. this finding was similar to the study done in northern Ethiopia and others [17,21]. This is because spontaneous delivery is less risky to mothers and mothers make them without trauma, with less general, and local anesthesia than forceps delivery. The study showed that mothers having 2-4 children delivered were 8.38 times [(AOR=8.38, 95%: (4.6, 15.3)] and mothers with ≥ 5 children delivered 18.2 times more likely [(AOR=18.2, 95%: (9.4, 35.4)] to practice kangaroo mother care than Prim-Para. This finding was similar to the study done in Aksum and Singapore [19,22]. This is because mothers have exposure to postnatal neonatal care, and they might have knowledge and awareness about kangaroo mother care.

Pregnant women 2-3 visiting antenatal care, were 3.29 times more likely [(AOR=3.299, 95% CI : (1.54, 7.07)] to practice kangaroo mother care than one-time visiting antenatal care. This finding was similar to the study done in Aksum and public health institutions of Ethiopia [19,20]. This is because mothers more time attending the antenatal care unit, can get counseling, and can know future neonatal care.

## Conclusion

The study assessed the magnitude of Kangaroo mother care practice in Tigray, Ethiopia, which is lower than the study done in Ethiopia-Amhara, and Uganda. Also, we identified that maternal age, parity, antenatal care, occupation, and mode of delivery were factors associated with kangaroo mother care practice, so healthcare providers, health organizations, and policy makers should give special focus on those factors to improve kangaroo mother care practice.

### What is known about this topic

- *Kangaroo mother care implementation is an evidence based practice can decrease preterm and low birth weight infant morbidity and mortality;*
- *There was lack of information on magnitude of Kangaroo mother care practice in the study area;*
- *Even though Kangaroo mother care practice is an evidence based practice that can decrease preterm and low birth weight infant morbidity and mortality, country-level adoption and practice of kangaroo mother care has been limited and coverage remains low*.

### What this study adds

- *About two-third participants was practiced Kangaroo mother care in the study area;*
- *Maternal age, parity, antenatal care, occupation, and mode of delivery were the significant factors associated with kangaroo mother car practice;*
- *Therefore, healthcare professionals must take on the promotion action by information, education and communication approach*.
